# Behavior of Defective Aluminum Panels Under Shear Forces Patched with Composite Plates—A New Engineering Approach

**DOI:** 10.3390/ma18174138

**Published:** 2025-09-03

**Authors:** Yuri Simanovskii, Haim Abramovich

**Affiliations:** Technion Faculty of Aerospace Engineering, Israel Institute of Technology, I.I.T., Haifa 32000, Israel; simyura@gmail.com

**Keywords:** damaged aluminum panels, composite patch, shear loading, finite element analysis, loading frame, experimental results, 3D elements, shear buckling

## Abstract

Old airplanes produced in the 1970s are still flying, while being exposed to various new types of detriments, leading to a need to repair them to enable the safe use of the airborne body. The present state of the art advocates the use of laminated composite to repair aluminum parts due to their effective durability. The studies presented in the literature mainly focused on bodies under tensile loads. It seems that shear-type loading appearing in the fuselage of airplanes when being under torsion has been ignored in literature. Therefore, to fill this gap, the present study investigates the behavior of defective aluminum panels under pure shear. The present investigation uses a novel finite element (FE) method of modelling the loaded body by 2D and 3D elements. Then, the model is used to calculate the influence of various parameters, like the size of the repair patch, overlaps, sequences of the laminated composite plate, and other structural properties on the stability and strength of the examined part. To validate the numerical predictions, tests were performed on typical elements. Based on the experimental results, the fidelity of the FE model was assessed and the method approach of repairing using composite patches was validated. The main conclusion from the present study is the use of solid (3D) elements, over shell (2D) elements, due to their high-fidelity results.

## 1. Introduction

### 1.1. Background

Old metal airplanes, produced in the early 1970s, are still flying, having new types of detriments, located in various parts of the airborne vehicle. Therefore, it is assumed that the aged metallic structure has lost its endurance, reaching the durability limit. As well as that, with non-careful maintenance, these would be the two main factors influencing the increase in maintenance. These air vehicles encounter external damage from different origins, such as Foreign Object Damage (FOD), non-careful maintenance, fatigue, etc. The Outer Mold Line (OML) of front or middle body sections usually consists of stress-carrying skins, subjected to torsion and reacted by shear flow load on the skins.

To perform a repair, its concept must be decided based on a damage evaluation that assesses whether it is within its allowable limits as defined by the manufacturer’s Structural Repair Manual (SRM). Five repair choices are available:

1. Leave it as it is—when the structural damage is insignificant from the static strength perspective.
2. A cosmetic repair—when the damage is insignificant from the static strength perspective, but the relevant part requires a refined aerodynamic finish.
3. Temporary repair—a damage repair that does not pose a problem to the structural durability of the relevant part, but it might lead to damage over time depending on the load.
4. Structural/permanent repair—when the damage threatens the immediate structural integrity (not only from the fatigue point of view), then a permanent fixture is required, usually by a repair patch applied on the damaged part.
5. Replacement—if the process of repairing the damaged component turns out to be not economically or workable, the damaged part should be substituted.

A metallic structure (skin type) may be repaired using two main methods—bolted or bonded repair. Both methods can use either composite or metallic parts. As a rule, a composite bonded repair would be favorite in comparison with bolted repair due to its better stress distribution and almost no stress concentration. However, the process for applying a composite bonded patch is more complex and attention to details would matter.

**Figure 1:**
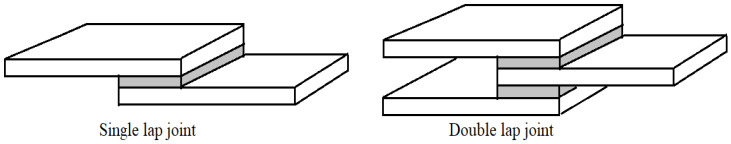
Bonded sole and dual segments.

**Figure 2:**
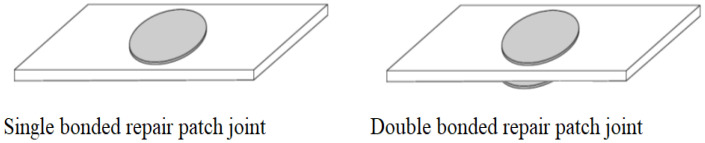
Sole and dual bonded repair patches [1].

Single lap joint approach is largely used in research studies and the industry to analyze glued parts (Figure 1). Some researchers investigate a patch repair type in their studies (Figure 2).

A sole bonded segment, as shown in Figure 1, would join two parts, called the “adherents”, with a layer of dedicated glue. The force applied on this joint is tensile axial (along the adherents), transferring fully from one adherent to another, subjecting the adhesive to shear load. It was found that a real glued repair part would only reinforce the damaged part. In an actual bonded repair, external patches are bonded to a damaged area to strengthen the defected part. Therefore, patch repair is more suitable for simulating an actual bonded repair. A repair patch concept transfers only a certain amount of load, and the rest bypasses the patch and creates stress concentration around it in the repaired adherent. The single lap joint does not consider stress concentration, supporting the fact that patch repair is more suitable to assess the ultimate stress of glued repair.

### 1.2. Literature Review

Patches, usually made of laminated composite materials, to restore airplane structures having cracks and/or other local defects have been widely used in the past few decades. Baker et al. [2] have shown that the use of laminated composite patches is the best economical way to extend the life service of old airplanes having local structural damage with acceptable structural effectiveness.

The method of bonded repair can be analyzed either using numerical approaches (like FEM, finite differences, or boundary elements) or using available closed-form solutions [3]. It was shown in [3] that in most cases, the glue is analyzed using a linear elastic model rather than the nonlinear model, which would lead to a more complicated analysis. In 1938, Volkersen [4,5] introduced a shear lag procedure to analyze glue bonded parts, which was assumed to behave linearly with the shear force leading to its deformation. Other factors, like peel stress, load eccentricity, and bending effects were ignored. Goland and Reissner [6] in 1944 introduced these missing factors in their study; however, the glue was assumed to behave linearly. Only in 1973 did Hart-Smith [7,8] present closed-form solutions for elastic–plastic adhesive, suggesting that the use of tapered adherents might reduce the local peel stresses and thus prevent dis-bonding of the parts.

It turned out that analyzing a bonded joint using a closed-from model is suitable only for the first stages of the structural design, as no failure criteria are incorporated in the procedure.

Therefore, computative approaches should be used, with the FEM being the most used numerical tool, to analyze bonded repairs due to their inherent capability to imitate force transfer in a complex structure.

The modelling of the glue layer is usually performed using shell and solid-type elements. In [9] the adhesive was modeled with solid elements to maximize the competence of a scarf joint, while [10] presents a single-lap joint analyzing with solid elements, within ABAQUS FE code based on an elastoplastic model. Contrarily to what is assumed in the literature, the research in [10] found a reasonable matching between computative and analytical solutions. Ref. [11] tackles the important issue of selection of element types to model laminated composite bonded repair, by presenting an interested comparative study.

Naboulsi and Mall [12,13], Schubbe and Mall [14], and Naboulsi and Mall [15] present interesting results for a computer model of composite and glue stacks, using a three-stack procedure in comparison with the 3D FE models. Later, successively adopted the three-layer procedure for a nonlinear analysis of the repaired structure taking in account large displacements and material nonlinearities.

An interesting review is presented by Da Silva and Campilho [16]. They found that almost all closed-form solutions for glued segments are 2D and assume linear behavior.

Rose [17,18] showed that the stress amplification factor range of a repaired structure does not depend on the crack length if the crack grows up below the repair. As a result, the crack growth rate does not depend on crack length, according to the Paris law.

Baker and Jones [19] present interesting results for damaged aluminum plates reinforced by laminated composite patches.

Achour et al. [20] presented a study on the use of FE analysis to model cracked plates with composite patches, confirming the results of older studies.

Rachid et al. [21] also analyzed cracked plates with bonded patches using 3D finite elements.

Wang and Rose [22] and Kumar and Hakeem [23] investigated the stress amplification factor (SAF) limit applied to cracked plates being reinforced by composite patches leading to similar results, as shown by Baker and Rose [2].

Another important aspect investigated was the fatigue issue. Klug et al. [24] presents results for the fatigue behavior of cracked 2024-T3 aluminum plates reinforced by carbon/epoxy patch. The fatigue life was increased by 4–5 times by using a one-sided reinforcement. A similar experimental study is presented by Okafor et al. [25], showing that there is a large decrease in skin stress due to the use of the repair patch. It is interesting to note that, in their research, Naboulsi and Mall [13] also predict the fatigue life of a repaired plate using a glued patch and compare it to computed stress amplification factor within the experimental results.

Finally, in more recent studies [26], composite patches were calculated to be used in civil engineering. Two reviews on improvements in repaired composites [27] and on parameters affecting the capability and endurance of composite patches [28] display the knowledge accumulated from literature. Application of failure analysis is presented in [29] with similar strength analysis described in [30]. The fatigue issue of structures being repaired by patches is presented in [31,32,33] (although the fatigue topic is not dealt with in the present study). Various optimum procedures to analyze damaged structures reinforced by sole and dual bonded composite patches using finite element analysis (FEA) and the Taguchi approach are presented in [34], with the team in [35] dealing with optimized patches based on strength analysis. In a 2025 paper [36], the approach of using composite patches with variable stiffness for single-sided bonded repair of composite structures under tensile loading is proposed with FEA results being validated by dedicated tests results. Another interesting 2025 paper [37] discusses the important issue of dis-bonding due to fatigue for models with cohesive zones, while in [38], Siciliani et al. address another critical issue of correctly preparing the surface of the CFRP compression molding laminates and using calibrated wires to obtain consistent bond thickness.

It is clear from the quoted literature review that the issue of applying composite patches to damaged aluminum plates under shear loads has not been dealt with and therefore the present study will provide new experimental and numerical insight into this topic. The present research aims at developing a better understanding of aircraft aluminum shear-load-transferring skin behavior, subjected to typical damage and recovered by composite material repairs. It consists of finite element simulations of the effects of stacking sequence, patch thickness, equivalent mechanical properties, and stiffness of the composite bonded repair patch, applied on an aluminum skin under shear load, subjected to typical damage. The FEA results are validated by experimental results yielding good matching.

## 2. Methods and Materials

### 2.1. The Finite Element Analysis (FEA)

**Figure 3:**
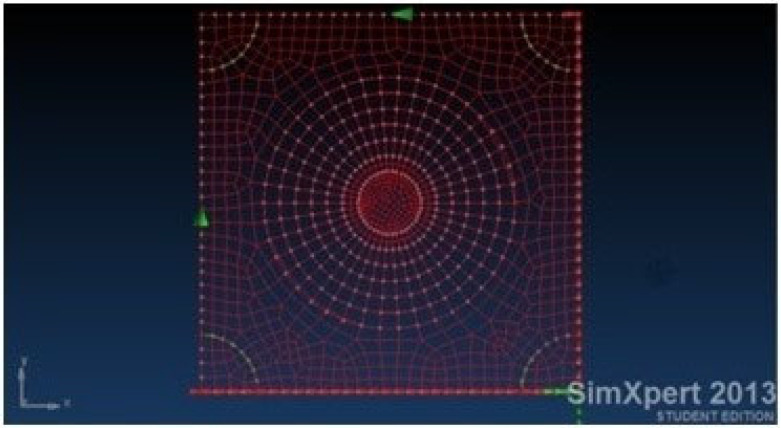
FEA model of the skin representing the chosen aircraft panel.

**Figure 4:**
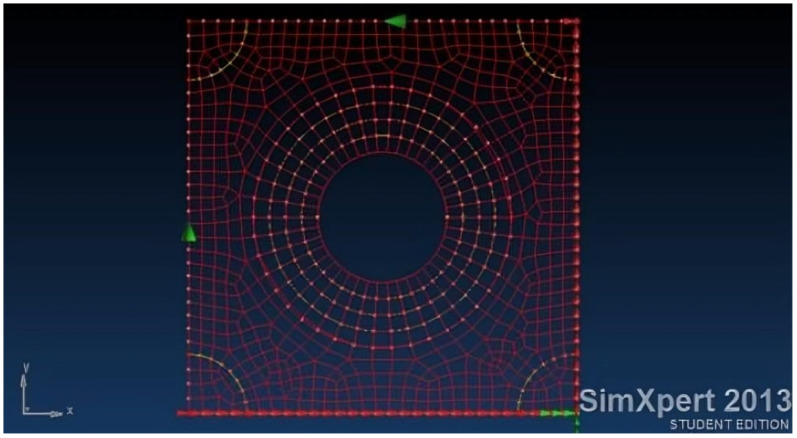
FEA model of the damaged skin having an 80 mm diameter hole (1/3 of the panel’s size).

Figure 3 presents a panel (modelled by finite element code SimXpert 2013 [39], see also Appendix A), representing an aircraft skin, subjected to pure shear load. Circular damage with 60, 80, and 100 mm in diameter is introduced as can be viewed in Figure 4.

**Figure 5:**
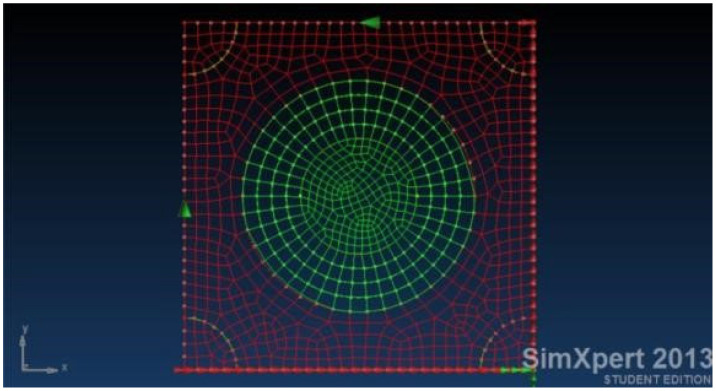
FEA model of the damaged skin with an 80 mm diameter hole and the single-sided repair patch (1/3 of the panel’s size).

Then a patch is modelled using isotropic/orthotropic shell elements, as described in Figure 5, while the connection between the panel and the patch is established using solid isotropic elements, representing the adhesive. At the first stage, all the patches are identically analyzed, having the same size (diameter)—no overlapping for the composite repair. The effect of the ply drop is further investigated for a chosen configuration, as an optimization stage.

Note that to develop the most adjacent repair (single-sided—defined as the main goal of the present study—repairing a panel without any complex disassembles on the aircraft; thus, a double-sided repair would be impossible), a preliminary analysis is performed—based on an aluminum single- and double-sided patch. This patch is designed to be 1.6 mm in width, the same as the panel, having a diameter of 120 mm and 140 mm—20 mm and 30 mm overlap beyond the damage size.

### 2.2. Materials and Properties

The material used to model the panel with isotropic shell elements is Aluminum 7075-T6, having the following mechanical properties: Young’s modulus E = 72.3730 GPa (7380 kg/mm^2^); shear modulus G = 27.4586 GPa (2800 kg/mm^2^); Poisson’s ratio ν = 0.3; ultimate tensile strength (UTS) σ_UTS_ = 0.54 GPa (55 kg/mm^2^); and ultimate shear stress τ_USS_ = 0.32362 GPa (33 kg/mm^2^).

The adhesive layer is assumed to be isotropic and is modelled using 3D solid elements to account for the geometrical eccentricity of the patch, relatively to the panel. The isotropic properties for the EA9396 adhesive are Young’s modulus E = 2.7557 GPa (281 kg/mm^2^); shear modulus G = 1.03 GPa (105 kg/mm^2^); Poisson’s ratio ν = 0.3; and allowed shear stress (ASS) τ_ASS_ = 0.02746 GPa (552.8 kg/mm^2^).

**Figure 6:**
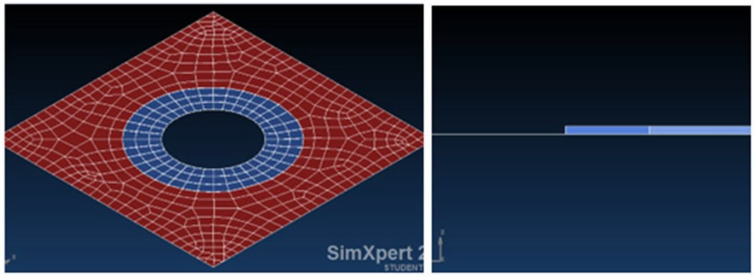
Finite element model of the adhesive area (in blue), thickness of adhesive 0.2 mm.

Note that during the manufacturing process, the glue smeared on the repaired area is pressed out using a vacuum process. The glue type used has low viscosity and is assumed to be approximately 0.2 mm, as described in Figure 6. Note that due to lack of adequate instruments, the adhesive bond line was not measured (see also [38] for reference). In the test program, the aluminum panel is taken to be Aluminum 7075-T0, and the laminated composite repair patches are made of carbon fabric 3K-70/Epoxy EA9396, prepared by wet layup. The bonding of the patch is achieved using Hysol EA9396 epoxy paste, from Henkel Corporation (Düsseldorf, Germany) [40] and widely used in the aerospace MRO (Maintenance and Repair Operations) industry. EA9396 has low viscosity and medium peel stress and cures at room temperature with very good strength properties. It can also be cured for one hour at 66 °C, as suggested by the manufacturer.

The patch is constructed of several composite layers (apart from the preliminary phase of this study, in which the patch was made from aluminum), with mechanical properties presented in Table 1. Note that the patch was modelled using composite shell element and E11 = E22, as the carbon fiber used was 3K-70 plain weave.

### 2.3. Boundary Conditions

**Figure 7:**
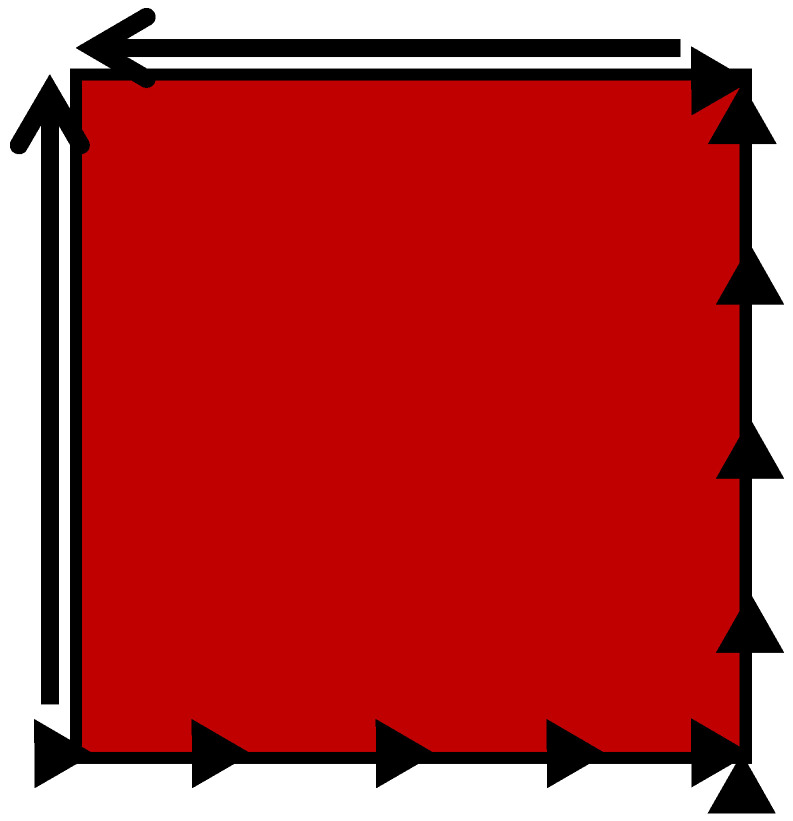
Schematic drawing of the skin representing the panel, subjected to load along two edges and constrained along two opposite edges, to yield pure shear load.

To subject the panel to pure shear load, two adjacent sides are loaded lengthwise, and the remaining sides are constrained, each in the load direction as the opposite side, plus in the out-of-plane directions. To fulfill the model’s requirements for sufficient constraint, a single node (in the corner in which constraint and loading are met) is chosen to be constrained in the load direction (and not only perpendicular and out of plane), as described in Figure 7.

### 2.4. Loading

Assuming that the maximum stress that can be achieved in the aluminum model, presented by the von Mises criteria, is 0.539 GPa (material ultimate allowable), the maximum allowed shear stress can be calculated using the following equation (see [41]):

τ_pureshear_ = σ_VM_/√3 = (0.539)/√3 = 0.31136 GPa (1)

where σ_VM_ is the von Mises stress.

Thus, the maximal applied load along the panel’s side may be obtained from the following equation:

F_all_ = τ_pureshear_·t·b = 0.31136 × 1.6 × 240 ≅ 119.573 kN (2)

### 2.5. Convergence of FEA Model

The convergence of the FEA is verified for the model described in Figure 4—the open-hole panel with an 80 mm diameter.

**Figure 8:**
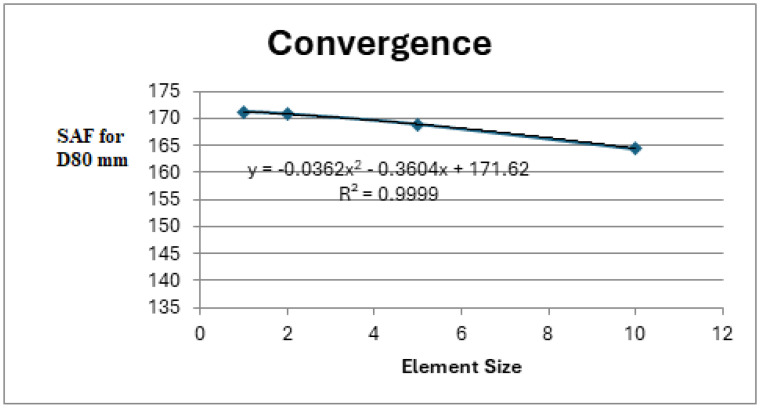
SAF for 80 mm damage in diameter vs. element size in finite element model to define convergence.

Figure 8 presents the convergence curve obtained for the stress amplification factor (SAF) in the hole vs. element size on the damage’s edge. As may be seen, the SAF increases for a denser mesh (smaller elements), though the difference between 10 mm elements and 2 mm elements is negligible (approximately 3%). Thus, 10 mm elements are used for the model, as there is no benefit in using smaller elements. Moreover, this research is mainly based on relative comparison; thus, the accuracy of the SAF is less important than its variability under influence of various patch repairs.

The convergence validation is relevant only for the open hole model, the same as for the other components—the adhesive and the patch are directly produced from the panel’s model, having a conforming mesh.

### 2.6. FEA of the Experimental Test Set-Up

**Figure 9:**
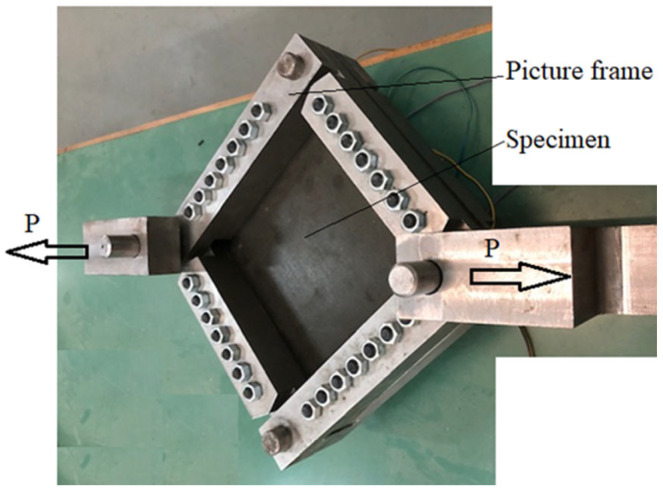
The test set-up used in the present study.

**Figure 10:**
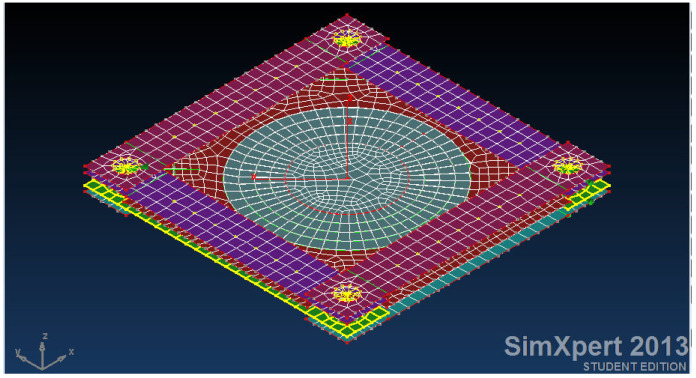
The FEA analysis of the test set-up.

The test set-up used in the present study is presented in Figure 9. To increase the accuracy of results, considering that the pure shear test presented in Figure 7 is only an ideal model, it was decided to simulate the test set-up using FEA, using 2D shell elements, as presented in Figure 10.

Note that the load is applied along the main diagonal (see Figure 9) and transferred to the tested specimen through the side frames.

**Figure 11:**
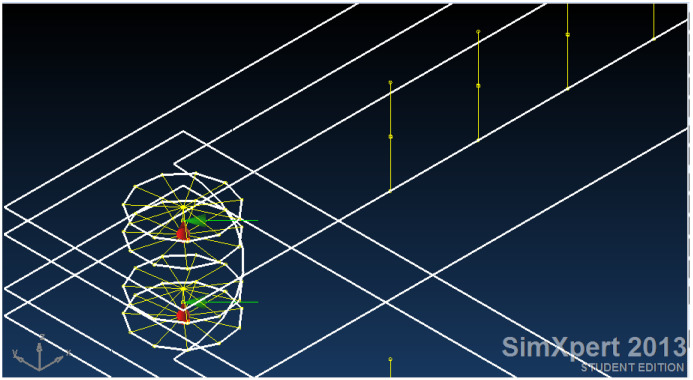
Bolt connection modelled as RBE2 elements between matching nodes and the bushings are also modelled with RBE2 elements based on spiders on the frame.

The frame is double-sided for symmetry and to account for out-of-plane bending. It is connected to the panel using 7 × d = 10 mm bolts on each edge and 4 free-of-friction bushings in the corners to redirect the applied tensile load in the direction of the main diagonal to compression load in the direction of the opposite diagonal. The bolt connections are modeled as RBE2 elements (see Appendix A) between matching nodes. The bushings are modeled as RBE2 as well; however, they are created as “spiders” on the bushing’s hole in the frame (Figure 11). Then, the spiders are connected by another RBE2 to define a rigid point for load application. The main difference between the RBE2 used for the bolts and the bushings is that the bushings allow for rotation around the Z-axis; thus, R3 (see Appendix A) is left open for bushing modeling.

The von Mises (or maximum stress theory) failure criterion was used in these FE models. Moreover, a linear buckling criterion is also examined to investigate stability of the structures, using the SOL105 approach.

## 3. Results

### 3.1. Study Cases

The cases studied in the present study were dictated by the dimensions of the experimental test set-up—the frame, which fits a 240 × 240 mm^2^ panel. Various damage sizes were chosen—25% of the panel’s length, 33.33%, and approximately 42%; for a more comfortable analysis, the damage was modeled to be circular.

Each case was analyzed using either single or double aluminum patches. Then, single and double composite patches were analyzed for various lay ups. The patches were chosen to have a 20 mm overlap beyond the damaged area. During this study, the patches were tapered at the edges; to investigate and optimize the tapering sequence, when required, the patches were oversized to compensate for loss in stiffness by tapering.

In the first stage, all analyses were investigated for static strength, and then all repairs and study cases were verified for buckling.

### 3.2. Aluminum Repair Patch Analysis Results

After performing a preliminary analysis (see Appendix C), an Aluminum 7075T6—0.81 mm double-sided d = 120 mm repair was chosen (half thickness based on standard aluminum sheet thickness).

**Figure 12:**
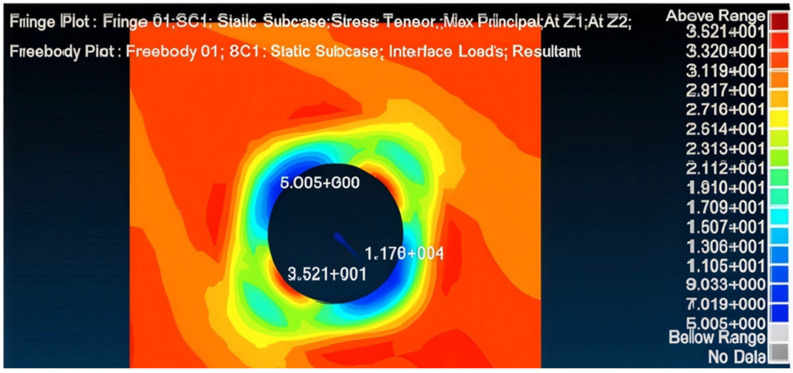
Maximal principal stress of the damaged panel for Aluminum 7075T6—0.81 mm double-sided (d = 120 mm) bonded repair.

**Figure 13:**
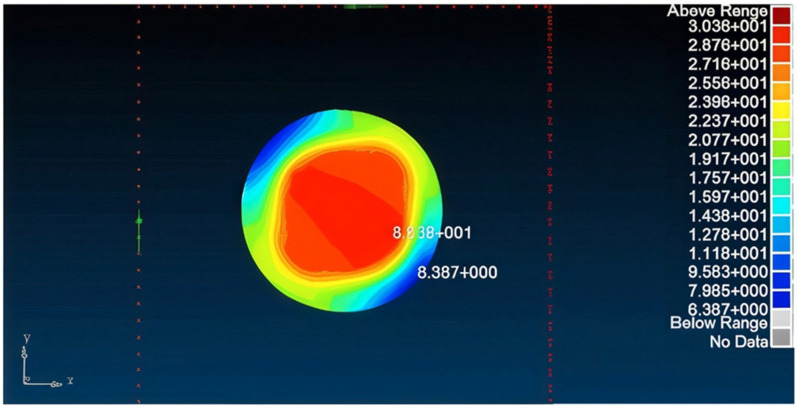
Max stress in load direction of Aluminum 7075T6—0.81 mm double-sided (d = 120 mm) bonded repair patch.

**Figure 14:**
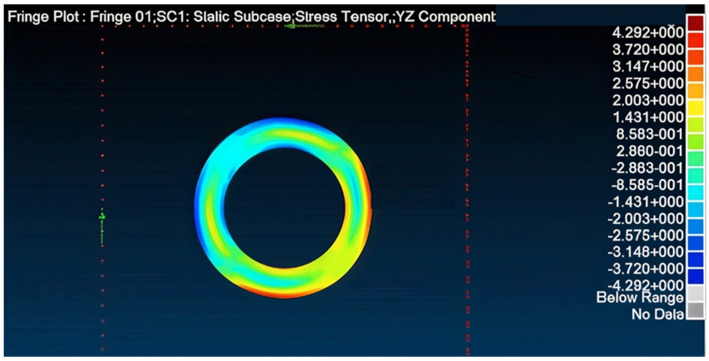
Max shear stress in load direction of the adhesive layer.

Application of such repair reduces the transferred load in the panel to 115.326 kN (Figure 12)—89% of the allowed load—also diminishing the SAF (stress amplification factor) to 1.11. Accordingly, the maximum principal stress obtained in the patches is 0.29773 GPa (Figure 13), smaller than the allowed stress. Similarly, the adhesive was verified to be able to sustain and transfer the applied loads and as can be seen in Figure 14, the shear stress amplification obtained at the edge of the bond line is higher than the allowed shear stress in the adhesive. To solve this issue, edge tapering was applied to the outer 10 mm ring of the patches to 0.42 mm (approximately 50% width deduction), leading to satisfactory results concerning the load transferred through the panel, shear stress in the repair patches, and the shear stress in the bond line edge, which were found to be smaller than allowed values.

### 3.3. Composite Repair Patch Design and Analysis Results

After careful examination and preliminary calculations, the chosen composite patch repair has 10 plies sequenced [45°, 0°, 0°, 0°, 45°]_s_ and tapered to 10 plies, followed by 6 plies and 2 plies with a nominal radius of 180 mm for a damage size of 80 mm. Note that the zero direction of the laminate overlaps the diagonal of the squared tested specimen.

**Figure 15:**
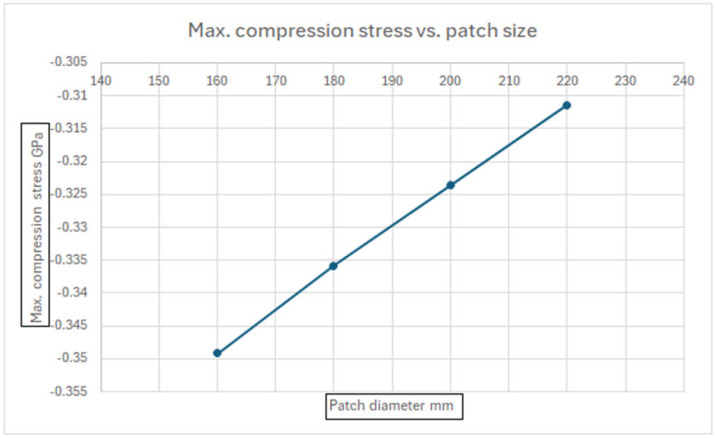
Maximal compression stress vs. patch size.

Figure 15 presents a linear correlation between the patch size (patch diameter) and the maximum compression stress in the composite patch. This figure allows for us to derive the required patch size for no failure to occur in the patch.

**Figure 16:**
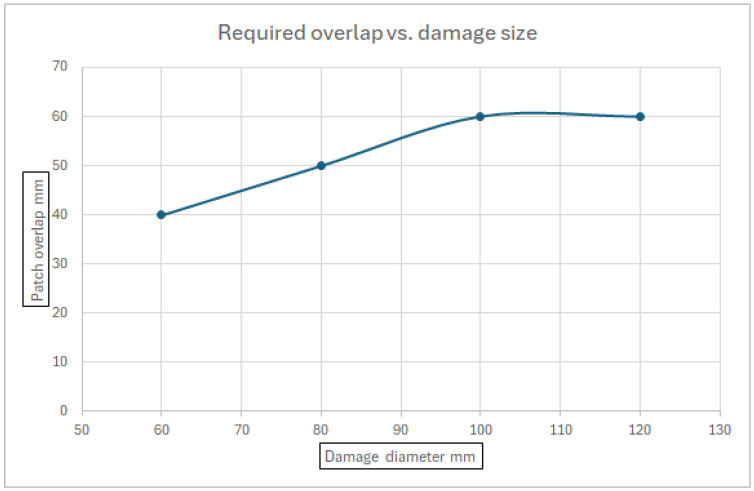
Maximal shear stress in the adhesive vs. patch size.

Figure 16 presents the relation between the patch size and the maximal shear stress in the adhesive—the larger the patch is, the smaller the stress in the adhesive.

Although a double-sided patch repair was found to provide a better strength solution, the present study concentrated on a single-sided patch due to inaccessibility issues in most aircraft.

**Figure 17:**
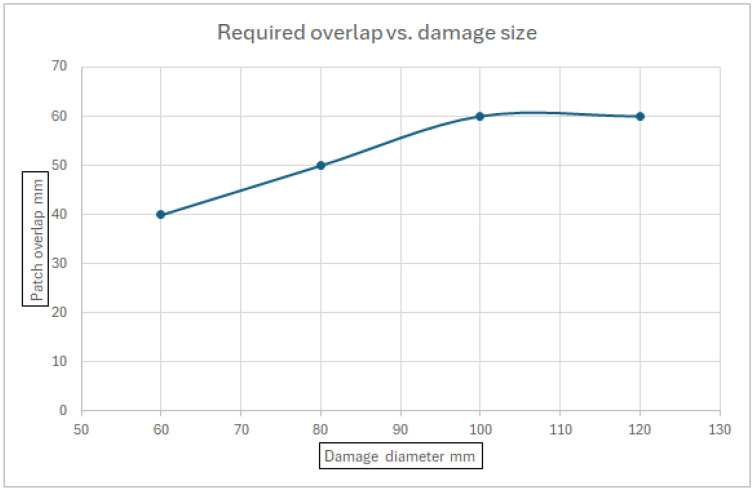
Required patch overlap vs. damage size.

Finally, a correlation between the damage diameter and the required overlap of the patch, for which no failure occurs under the applied loads, is shown in Figure 17. As can be obtained from Figure 17, the theoretical maximum effective overlap is around 60 mm.

- Figure 17—Required patch overlap vs. damage size

One should note that although a double-sided patch repair was found to provide a better strength solution, the present study concentrated on a single-sided patch due to inaccessibility issues in most aircraft.

### 3.4. Buckling Analysis Results

To calculate the theoretical buckling load for a squared simply supported isotropic plate under pure shear, the Timoshenko [29] equation is used:

(3) $$\tau_{cr}=k_{s}\cdot \frac{\pi^{2}\cdot E}{12\cdot (1-\vartheta^{2})}\cdot {\left(\frac{t}{b}\right)}^{2}$$

The shear buckling coefficient k_s_ is found to be 9.34. Then, substituting the relevant data for aluminum yields the critical shear stress for buckling:

(4) $$\tau_{cr}=k_{s}\cdot \frac{\pi^{2}\cdot E}{12\cdot \left(1-\vartheta^{2}\right)}\cdot {\left(\frac{t}{b}\right)}^{2}=9.34\cdot \frac{\pi^{2}\cdot 72.373}{12\cdot \left(1-0.33^{2}\right)}\cdot {\left(\frac{1.6}{240}\right)}^{2}=0.02773\;GPa$$

where E and ϑ are the Young’s modulus and Poisson’s ratio of the plate, respectively, and t and b are the thickness and width of the plate, respectively.

Multiplying the shear critical stress by the side area of the specimen (240 × 1.6 mm^2^) gives a shear load of 10.65 kN, yielding the tensile load of 15.053 kN to be applied on the shear frame.

Note that the shear buckling load is much lower than the stress obtained from strength calculation (0.31136 GPa) and the buckling issue will be the main factor to determine the correct type of the repair patch.

**Figure 18:**
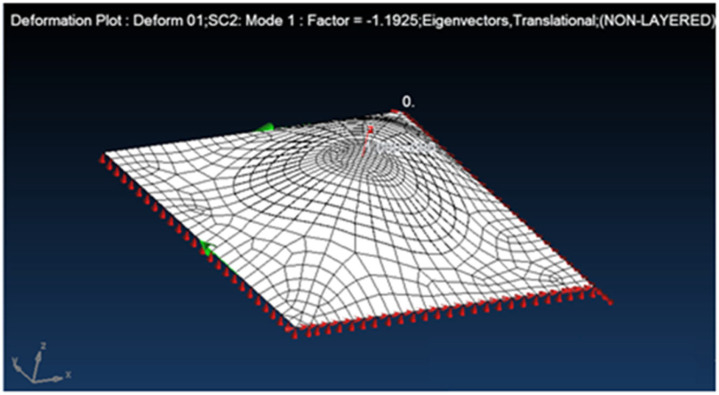
FEA buckling mode: a single-sided aluminum patch bonded repair applied to the damaged panel.

At the first stage, we use an aluminum patch, like the panel original material, to repair the hole. The result obtained for a d = 160 mm patch is a buckling factor (BF) of 1.1925 (see Figure 18) (a buckling factor BF = 1 would be the for the panel without repair), which indicates that an aluminum patch repair increases rigidity more than required. Using a smaller patch, d = 120, yields a much smaller BF of 1.0943.

Next, a laminated composite patch is designed and calculated for various layups. The results are summarized in Table 2.

**Figure 19:**
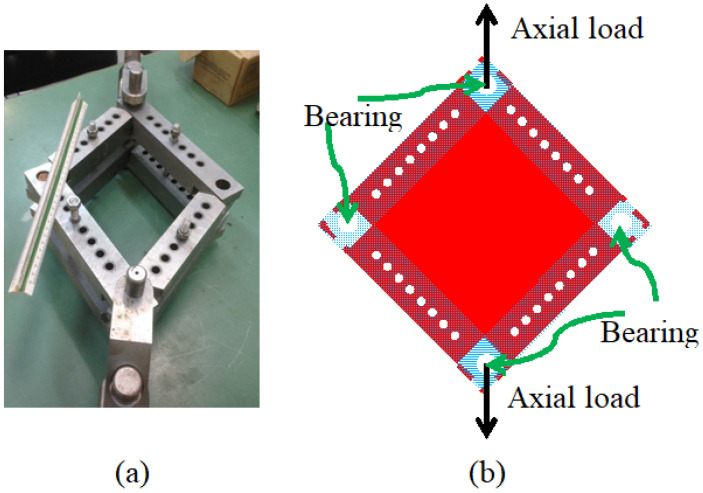
(**a**) The loading frame; (**b**) schematic drawing of the loading frame.

Note that in Table 2, the 0° direction coincides with the direction of the tensile load applied on the picture frame (see Figure 9 and Figure 19 for reference) with the 45° directions being along the sides of the square plates.

It is evident from Table 2, that the best configurations for BF restoration are [45°, 0°, 0°, 45°, 0°, 0°, 45°], [0°, 45°, 45°, 45°, 45°, 45°, 0°], and [45°, 0°, 45°, 0°, 45°, 0°, 45°]. The last one is the most exact result, while the first two are approximately 1–2% more rigid.

### 3.5. Test Program and Results

To apply pure shear test on a panel, using a single axis test platform, a “Frame” adopter is required to mechanically transform tension load to shear load along the panel’s edges, as described in Figure 19.

The tested specimens were instrumented with triaxial staring gages from KFG series from KYOWA-Tokyo, Japan [42].

The predicted results for the four specimens tested during the test program are summarized in Table 3.

#### 3.5.1. Original Panel and Panel with a Hole Test Results

**Figure 20:**
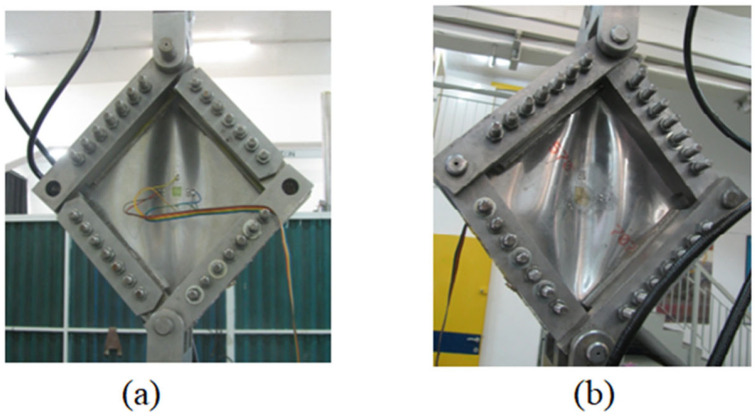
(**a**) Initial shear buckling mode; (**b**) collapse mode [43].

First, the original panel, with no damage, was tested. It started the initial buckling at approximately 16.20 kN (compared to the predicted value of 14.71 kN) (see Figure 20a) and leading to a collapse mode at 31.0 kN (compared to the predicted value of 29.116 kN) (see Figure 20b). Note that introducing a hole with 80 mm diameter leads to a collapse mode at 18.161 kN, which is 58.6% of the collapse load for an undamaged panel, as expected.

#### 3.5.2. Composite Patch Repair [0°, 45°, 45°, 0°] Test Results

The experimental shear buckling load was found to be 10.5 kN compared with the predicted value of 10.10 kN (see Table 3). The collapse happened at approximately 29.645 kN compared to 29.12 kN (see Table 3).

The comparison of the strain results at a load of 10.709 kN is displayed in Table 4.

As presented in Table 4, the experimental results are in good correlation with the predicted ones, except for the repair in the y-direction. The reason for this discrepancy might be the lack of smoothness due to inappropriate sealing of the damage noticed during testing.

#### 3.5.3. Composite Patch Repair [45°, 0°, 0°, 45°] Test Results

The experimental shear buckling load was found to be 10.72 kN compared with the predicted value of 9.954 kN (see Table 3). The collapse happened at approximately 30.51 kN compared to 29.12 kN (see Table 3).

The comparison of the strain results at a load of 10.502 kN is displayed in Table 5.

For this patch configuration (see Table 5), the experimental results show very good correlation with the predicted ones.

## 4. Discussion

The aim of the present research was to obtain a better understanding of aircraft aluminum shear-load-transferring skin behavior, subjected to typical damage and recovered by composite material repairs. Both experimental tests and a numerical study, based on FEA, were performed during this research. Experimental test results were used to validate accuracy of the parametric study by a finite element model to investigate the performance of wet layup patch repair. The effects of patch stacking sequence and sizes were parametrically studied using FEA models. The following outcomes are the conclusions from the present study:

- Metallic panels under shear load must be verified for both stress and buckling. As for buckling, it depends directly on geometry and less on strength properties; this issue must be first verified. Nevertheless, from the performed analysis, it may be concluded that designing a composite repair patch for an actual aircraft panel (heat-treated, having higher strength properties as opposed to annealed O treatment) covers the design requirements for buckling. In our analysis, verified by an experiment, we encountered the buckling phenomena due to usage of untreated aluminum.
- Parametrically studying the effect of the ply application sequence, based on analysis and verified in the experiment, it was found out that application of a ply at 0° (along load application) decreases the stress amplification factor on the damage edge, allegedly enabling later failure, compared to a patch, applied at 45° to the applied load direction. Corresponding to the previous statement, the experiment demonstrated buckling later (higher load of buckling) for the patch applied in the load direction (0°). Thus, 0° plies should be used when adding extra repair plies to increase carrying load capability.
- A parametric method, based on a finite element model, designed by a new approach of connecting shell elements with 3D elements, was performed. Based on the performed tests on four representative specimens, the finite element model was substantiated in all sampling points of buckling load and strain measurement locations. It was found that the finite element model is forever conservative by a maximum of 10% apart from only one strain measurement of one of the composite patches. In that patch, a lack of smoothness may be noticed due to insufficiently proper sealing of the damage.
- As a result of analysis validation, it may be concluded that a wet layup patch repair effectively restores the loss of strength caused by structural damage. The best solution would be to bond patches at both sides of the damaged plate; however, the approach presented throughout the present study to apply a single patch due to operational access constraints to the damaged plate was shown to be feasible both numerically and experimentally.

## Figures and Tables

**Table 1:** Carbon 3K-70/Epoxy EA9396 mechanical properties.

Property	Notation	Carbon 3K-70/Epoxy EA9396	
Main Young’s modulus	E_11_	4995 [kg/mm^2^] ^1^	48.98 [GPa]
Minor Young’s modulus	E_22_	4995 [kg/mm^2^]	48.98 [GPa]
Main Poisson’s ratio	$\upsilon_{12}$	0.08	0.08
Allowable tensile stress in the 11 dir.	$\sigma_{t11}$	45 [kg/mm^2^]	0.44 [GPa]
Allowable tensile stress in the 22 dir.	$\sigma_{t22}$	45 [kg/mm^2^]	0.44 [GPa]
Allowable compression stress in the 11 dir.	$\sigma_{c11}$	32 [kg/mm^2^]	0.314 [GPa]
Allowable compression stress in the 22 dir.	$\sigma_{c22}$	32 [kg/mm^2^]	0.314 [GPa]

^1^ Throughout the present manuscript the MKpS unit system in parallel with the equivalent SI unit system is used because the material properties are given by the manufacturer using the MKpS unit system. Note that 1 kg/mm^2^ = 0.00980665 GPa.

**Table 2:** Summary of the laminated patch results for different sequences and plies.

No of Plies	Lamination Sequence	d_hole_ = 80 mm	BF
0	-------	No patch d = 0 mm	0.506
3	45°, 0°, 45°	d = 160	0.598
3	0°, 45°, 0°	d = 160	0.614
3	45°, 45°, 45°	d = 160	0.573
3	0°, 0°, 0°	d = 160	0.6166
4	45°, 0°, 0°, 45°	d = 160	0.676
4	0°, 45°, 45°, 0°	d = 160	0.6935
4	45°, 45°, 45°, 45°	d = 160	0.6246
4	0°, 0°, 0°, 0°	d = 160	0.7125
5	45°, 0°, 0°, 0°, 45°	d = 160	0.77345
5	45°, 0°, 45°, 0°, 45°	d = 160	0.7593
5	0°, 45°, 45°, 45°, 0°	d = 160	0.7805
5	0°, 45°, 0°, 45°, 0°	d = 160	0.7955
5	45°, 45°, 45°, 45°, 45°	d = 160	0.6958
5	0°, 0°, 0°, 0°, 0°	d = 160	0.8193
7	45°, 0°, 45°, 0°, 45°, 0°, 45°	d = 160	1
7	45°, 45°, 0°, 0°, 0°, 45°, 45°	d = 160	0.9822
7	0°, 45°, 45°, 0°, 45°, 45°, 0°	d = 160	1.03
7	0°, 45°, 0°, 45°, 0°, 45°, 0°	d = 160	1.0459
7	0°, 0°, 45°, 45°, 45°, 0°, 0°	d = 160	1.0612
7	45°, 0°, 0°, 45°, 0°, 0°, 45°	d = 160	1.0177
7	45°, 0°, 0°, 0°, 0°, 0°, 45°	d = 160	1.0259
7	0°, 45°, 0°, 0°, 0°, 45°, 0°	d = 160	1.0542
7	0°, 0°, 45°, 0°, 45°, 0°, 0°	d = 160	1.0694
7	0°, 45°, 45°, 45°, 45°, 45°, 0°	d = 160	1.011
7	45°, 0°, 45°, 45°, 45°, 0°, 45°	d = 160	0.9858
7	45°, 45°, 0°, 45°, 0°, 45°, 45°	d = 160	0.9689
7	45°, 45°, 45°, 45°, 45°, 45°, 45°	d = 160	0.8968

**Table 3:** Predicted strains and buckling and collapse results for the experiments.

Test #	Hole Diam. [mm]	No of Repair Plies	Orientation	Buckling Load [kN]	Strain [μ] at Edge of Damage (x,y)		Strain [μ] at Center (Panel or Repair) (x,y)		Collapse Load [kN]
1	0	0	---------	14.71	507	507	507	507	29.11
2	80	0	---------	7.63	238	188	---------	---------	26.23
3	80	4	[45°, 0°, 0°, 45°]	9.954	394	288	665	666	29.12
4	80	4	[0°, 45°, 45°, 0°]	10.10	417	316	670	674	29.12

**Table 4:** Comparison of experimental strain results with predictions at 10.709 kN.

	Panel [x Dir.]	Panel [y Dir.]	Repair [x Dir.]	Repair [y Dir.]
Test [μs]	378.5	311	664.5	516.5
Prediction [μs]	417	316	670	674
Error [%]	9.2	1.6	1	30

**Table 5:** Comparison of experimental strain results with predictions at 10.502 kN.

	Panel [x Dir.]	Panel [y Dir.]	Repair [x Dir.]	Repair [y Dir.]
Test [μs]	370.5	286.5	649	649
Prediction [μs]	394	288	665	666
Error [%]	6.3	0.5	2.5	2.6

**Figure A1:**
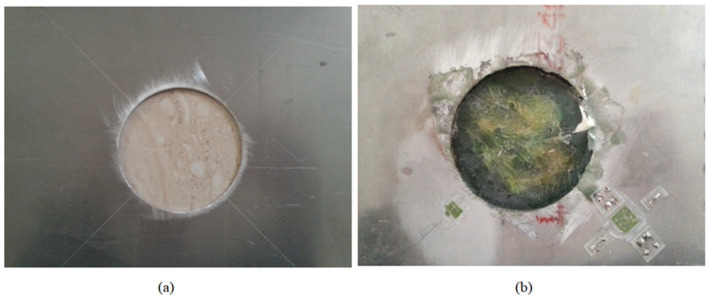
(**a**) Before sealing; (**b**) after sealing the damage with adhesive for vacuum process.

**Figure A2:**
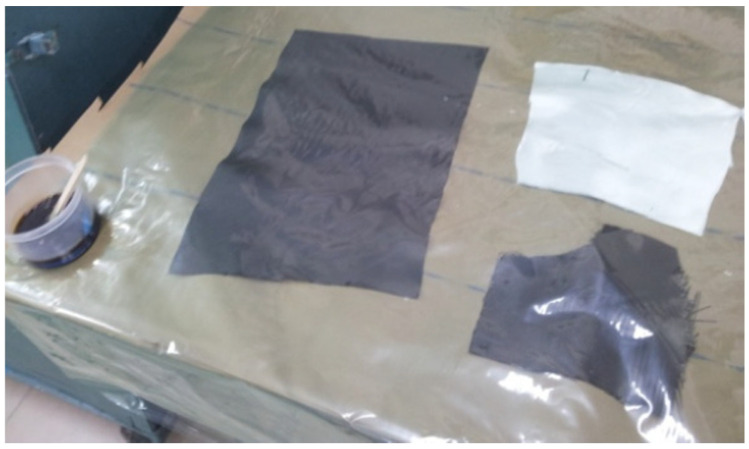
Dry carbon and glass fabrics placed on a polyethylene sheet, before wet layup impregnation.

**Figure A3:**
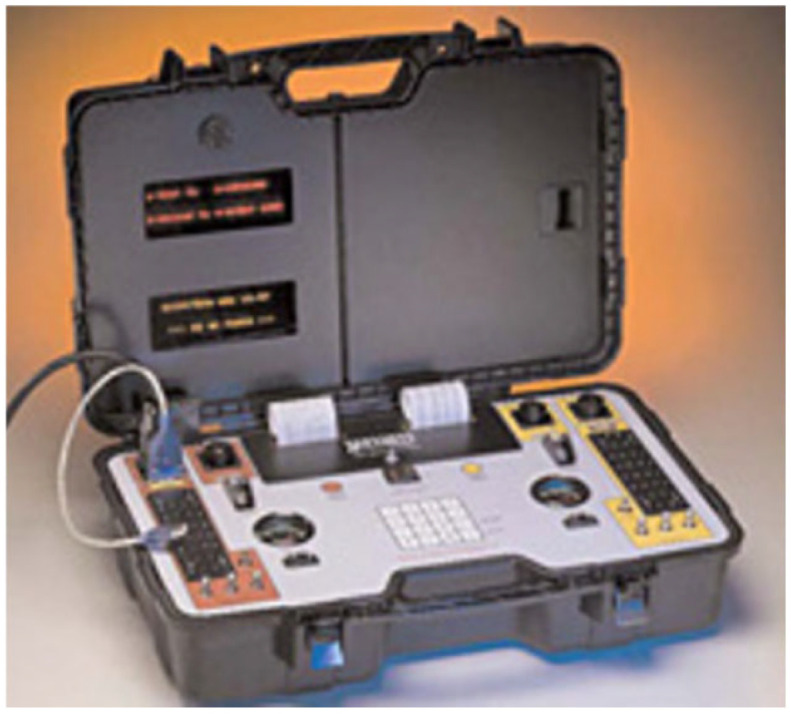
The HB-2 device for controlling vacuum and heat for accelerated curing process.

**Figure A4:**
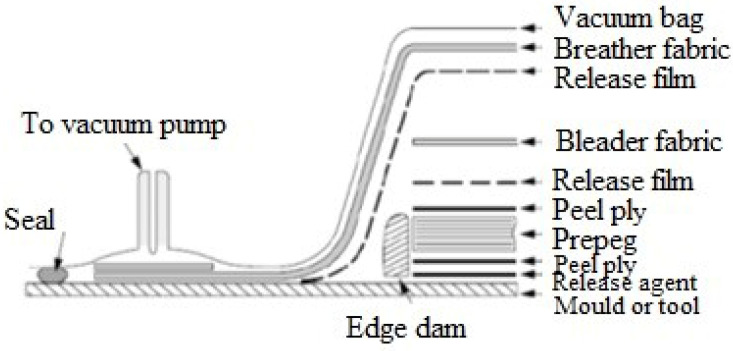
Schematic stacking sequence of various plies as a part of the vacuum bagging.

**Figure A5:**
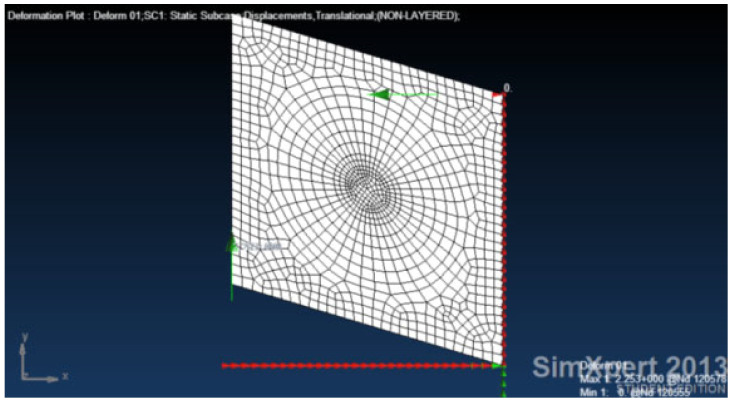
Finite element deformation of the panel, subjected to loads along the panel’s sides. The deformation of a parallelogram is a representation of pure shear.

**Figure A6:**
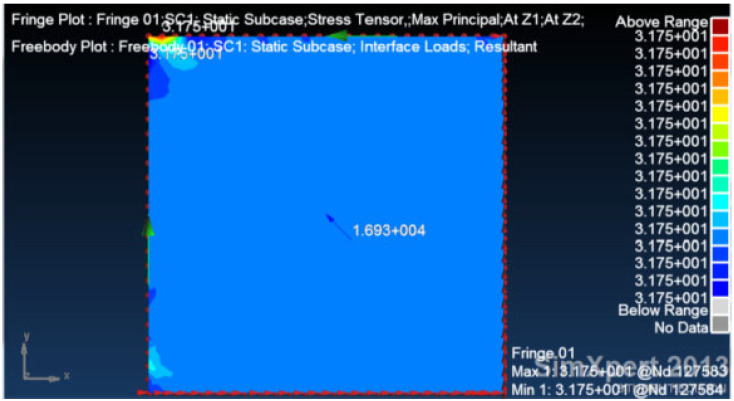
Finite element max principal fringe of the panel, subjected to loads along the panel’s sides. The uniform stress is a representation of pure shear.

## Data Availability

The original contributions presented in this study are included in the article. Further inquiries can be directed to the corresponding author.

## Abbreviations

3D	Three-dimensional
2D	Two-dimensional
AL	Allowed
BF	Buckling factor
FEM	Finite element method
FOD	Foreign Object Damage
OML	Outer Mold Line
MRO	Maintenance and Repair Operations
R3	Three-point method in SimXpert 2013 code
RBE2	Rigid body elements in SimXpert 2013 code
SAF	Stress amplification factor
SG	Strain gage
SRM	Structural Repair Manual
UTS	Ultimate tensile stress
USS	Ultimate shear stress
VM	von Mises

## Appendix A

SimXpert 2013 code was developed by MSC Software and is a unified CAE (Computer-Aided Engineering) environment designed for product simulation. It integrates multidisciplinary analysis capabilities, best simulation methodologies, and customization options to accelerate simulation speed and accuracy, enhance design productivity, and facilitate faster product development. The code allows for various types of analyses, including structural, motion, explicit, and systems and controls, within a single platform.

RBE2 elements, also known as rigid body elements, are used to create rigid connections between nodes. They effectively enforce rigid behavior between a single independent node and multiple dependent nodes, meaning these nodes move together as a rigid body. RBE2s are useful for modelling connections, applying fixed boundary conditions, and representing rigid connections in a finite element model.

R3 refers to the three-point method for defining a Cartesian coordinate system, specifically within the Structures Workspace. This method is used to establish a new local coordinate system by defining three points on a part. These points determine the origin, X-axis, and XY plane of the new coordinate system.

## Appendix B. Specimen Manufacturing

### Appendix B.1. Filler Insertion

The first stage is to fill in and stuff the damage to achieve a vacuum, later in the process of repair. The filler has no structural importance; thus, any filling process is acceptable. In our study, the hole was filled using an adhesive, as presented in Figure A1.

### Appendix B.2. Surface Preparation

Surface preparation is the first step to achieving a good bonding between two parts. Therefore, sanding, cleaning, and etching are to be carried out to create good adhesion in the bonding area.

*Sanding*—To prevent the deterioration the bonding effect, the surface must be polished using a coarse abrasive paper, 180-grit. There are two main sanding tools, namely, abrasive paper and power sander. In the present study, both methods were applied depending on the circumstances.

*Cleaning*—The damaged panel is thoroughly cleaned up using a solvent wash of Methyl Ethyl Ketone (MEK)/Acetone to remove contaminants on the glueing area.

*Etching*—One of the most common ways for aluminum surface activation in case of adhesion is using a “SEMCO Pasa-Jell 105”—a balanced blend of acids, activators, and inhibitors that acts as an etching material. By etching, it removes the top contaminated layer and uncovers a “pure”—high potential—aluminum, ready for intermolecular connection.

### Appendix B.3. Surface Preparation QA (Quality Assurance)

The acid is from the previous action is then removed using purified water, conducting a Water Break Free test to examine the bonding surface. If the water film easily flows, then the surface is ready for glueing.

### Appendix B.4. Plies Fabrication

The dry fabric roll (see Figure A2) is then cut into predefined dimensions. EA9396 resin is prepared with a mixing ratio of 100/30 (Part A/Part B—hardener) by weight. Once the resin is ready, bagging film is laid on a flat working plate and a dry fabric is placed on the film (Figure A2). Next, the mixed resin is shed on the dry fabric to saturate it. Using a spatula and a brush, the resin is evenly impregnated on the dry fabric.

Then, another layer of bagging film is placed on top of the impregnated fabric. The fabric is then squeezed (through the bagging film) to make sure the dry fabric is fully saturated and there are no excessive residues of resin. Using prefabricated paper molds, ply dimensions are marked on the bagging film and cut out using scissors. Then, the bagging films are removed, and the wet layers are placed axi-symmetrically on top of the filler.

### Appendix B.5. Curing Process

There are two curing specifications for EA9396, as presented by the material supplier: curing the wet layup at 25 °C for 3–5 days, or accelerating the process by curing at 66 °C for one hour or at 82 °C for half an hour. The shortest process was adopted for curing the specimen, using an HB-2 (HB-2 device is manufactured by WICHITECH industries Inc. (Randallstown, MD, USA) https://www.wichitech.com/prod/classification/hot-bonders/hb-2-composite-repair-system/) (accessed 14 July 2017) device (Figure A3), which provides controlled heating using thermocouples.

To perform the curing process, vacuum bagging must be used, as schematically described in Figure A4, while vacuum is being measured by the HB-2 device.

## Appendix C

Prior to the main part of this study, a conceptual analysis process for a d = 80 mm hole in an aluminum panel (7075T6-t = 1.6 mm) was performed. The material properties of the aluminum mentioned in Section 2.2 indicate that the ratio between the allowed tensile and shear stress is smaller σ_VM_$\;=\sqrt{3}$τ_USS_ (σ_UTS_/τ_USS_ = 0.54/0.32362 = 1.67 < $\sqrt{3}$).

Thus, for this specific case, limitations were derived by VM stresses, allowing for calculation of the allowed shear load along the edge, namely, τ_AL_ = σ_VM_/$\sqrt{3}$= 0.54/1.32 = 0.31177 GPa.

Considering the area of the edge to be 1.6 × 240 = 384 mm^2^ = 384 × 10^−6^ m^2^, we find the allowable shear load to be 0.31177 × 384 × 10^−6^=119.723 kN.

Consequently, the allowed tensile load to be transferred in diagonal direction is 119,577.4$\sqrt{2}$ = 169.315 kN.

When a load of 119.572 kN is applied on two sides of the panel, creating pure shear loading, the deformation obtained from the model presented in Figure A5 and the stress map obtained (maximum stress principle) is uniform and correlates to the calculated value of τ_AL_ = 0.31177 GPa, as described in Figure A2. Moreover, Figure A6 presents the load transferred through the diagonal—166.026 kN. This calculated load is a 1.82% negligible deviation from the calculated value. At this stage, it is possible to calculate the allowed load to be transferred to the panel, assuming a d = 80 mm hole:

$$\left(L_{diagonal}-d_{hole}\right)\cdot t_{plate}\cdot \tau =\left(340-80\right)\times 1.6\times 0.31177=129.696\;kN$$

It may be noticed obviously that if no repair patch is applied, the panel will not hold under the original load due to a major decrease in the panel’s cross-section.
